# Zinc Oxide Nanostructure Deposition into Sub-5 nm Vertical Mesopores in Silica Hard Templates by Atomic Layer Deposition

**DOI:** 10.3390/ma17102272

**Published:** 2024-05-11

**Authors:** Tauqir Nasir, Yisong Han, Chris Blackman, Richard Beanland, Andrew L. Hector

**Affiliations:** 1 School of Chemistry, University of Southampton, Highfield, Southampton SO17 1BJ, UK; t.nasir@soton.ac.uk; 2 Department of Physics, University of Warwick, Coventry CV4 7AL, UK; yisong.han@warwick.ac.uk (Y.H.); r.beanland@warwick.ac.uk (R.B.); 3 Department of Chemistry, University College London, London WC1E 6BT, UK; c.blackman@ucl.ac.uk

**Keywords:** atomic layer deposition, mesoporous silica, zinc oxide, small pores, electrochemically assisted surfactant assembly

## Abstract

Nanostructures synthesised by hard-templating assisted methods are advantageous as they retain the size and morphology of the host templates which are vital characteristics for their intended applications. A number of techniques have been employed to deposit materials inside porous templates, such as electrodeposition, vapour deposition, lithography, melt and solution filling, but most of these efforts have been applied with pore sizes higher in the mesoporous regime or even larger. Here, we explore atomic layer deposition (ALD) as a method for nanostructure deposition into mesoporous hard templates consisting of mesoporous silica films with sub-5 nm pore diameters. The zinc oxide deposited into the films was characterised by small-angle X-ray scattering, X-ray diffraction and energy-dispersive X-ray analysis.

## 1. Introduction

In recent years, nanostructured materials have attained significant prominence in various research communities due to their unique physical and chemical properties. The advantageous properties of nanostructured materials makes them an excellent choice compared with their bulk counterparts in various fields such as sensing, catalysis, energy storage, devices and biomedical applications [1,2]. These nanostructures can be divided into four categories based on their dimensions, i.e., (a) zero-dimensional nanostructures have all the dimensions in nanoscale (e.g., nanoparticles), (b) one-dimensional when only one of the three dimensions is above the nanometer range (e.g., nanowires and nanotubes), (c) two-dimensional nanostructures with two dimensions above the nanometer range (e.g., nanosheets and thin films) and (d) three-dimensional nanostructures with all three dimensions larger than 100 nm but nanoscale structuring within the bulk material (e.g., nanoflowers, three-dimensional network structures and nanowire bundles) [3]. Various strategies have been developed for the synthesis of nanostructured materials, which can be broadly categorised as top-down and bottom-up techniques [4]. Top-down techniques mostly rely on physical means, such as physical vapour deposition followed by lithography for nanostructure synthesis. On the other hand, bottom-up techniques consist of chemical and biological means and build up the nanostructures from the precursor materials [5]. Bottom-up techniques are most commonly used to produce nanostructures and consist of methods such as electrodeposition, chemical vapour deposition, sol-gel methods and chemical co-precipitation methods [6].

Templated synthesis is a sophisticated process used to fabricate nanomaterials of varying sizes and shapes. The porous structure of a host template is used to direct the growth of the material of interest. This technique that is used to produce nanostructures was first introduced by Possin, and further modifications were made by others [7,8]. Templating can be divided into two categories, i.e., hard and soft templating. Soft templating is based on cooperative assembly between the surfactant and the inorganic phase. The process is driven by “self-assembly”, where amphiphilic molecules self-assemble in a solution and combine with reaction precursors to form composite structures [9]. Hard templating, also known as nano casting, is based on filling or coating the rigid porous template with a desired material to form uniform nanostructures. Hard templating is desirable for the synthesis of nanostructures, as it excludes the need to control the hydrolysis and condensation of desired materials. It can produce a variety of crystalline structures with different sizes and morphologies. The challenges associated with the hard-templated synthesis of nanostructures are control over growth, incomplete filling of pores due to slower mass transfer and inevitable growth of precursor materials on the surface of template [10]. A commonly used hard template for the synthesis of nanostructures is anodic alumina membranes produced via the electrooxidation of high purity aluminium in acidic media. These membranes have pore sizes ranging from 10 nm to 2 µm, but are almost always produced above the 50 nm pore size. Track-etched polymer membranes are another type of hard template with pore sizes ranging from 10 nm to 10 µm, but again, the pore sizes are typically >50 nm. These templates are produced by tracking a film of polymer with high-energy particles followed by etching of the tracks with an alkaline etching solution. Polyethylene terephthalate (PET), polycarbonate (PC) and polyimide (Kapton) are a few examples of polymeric materials used to produce track-etched polymer membranes [11]. Mesoporous silica films are used less often as hard templates for the synthesis of nanostructures due to the challenge of depositing and characterising materials in the size range of their pores. These templates have pore sizes ranging from 2 nm to 10 nm with 1D and 2D pore alignment. These mesoporous silica films are produced using sol-gel self-assembly processes and synthesised mainly by dip coating and spin coating [5].

There are various techniques used for template filling or the synthesis of nanostructures using hard templates. These methods include electrodeposition and electroless deposition, melt and solution filling, lithography, layer-by-layer deposition and vapour deposition. Electrodeposition involves the application of an external potential that results in the reduction of cations in the solution, causing them to deposit in/on the template. Melt and solution filling are also used to produce metallic nanowires. Melt filling is based on injecting the liquid into porous templates using the pressure injection technique, whereas solution filling involves immersing the porous templates in a precursor solution, often followed by other treatments and reduction under H_2_ flow [12,13]. Lithographic techniques can be used to produce highly repeatable, uniform and high-yield nanostructures. Layer-by-layer deposition is a popular technique for nanostructure synthesis and is often considered to overlap the electrodeposition and solution filling-based techniques. The final shape and size of the desired material depends on the template structure, so the affinity and compatibility are the most important factors to consider while using layer-by-layer deposition [14]. Vapour deposition is another versatile and highly efficient technique that is used to deposit nanostructures and includes techniques such as chemical vapour deposition, plasma vapour deposition and atomic layer deposition.

Atomic layer deposition (ALD) is a powerful technique widely used to deposit thin films and nanostructures. The ALD process uses a sequential exposure of gas-phase precursors to deposit ultra-thin conformal coatings with high precision and reproducibility, and it has been widely used for nanomaterial synthesis, e.g., for catalysts [15]. Owing to its excellent reproducibility and control at molecular level, ALD can be used to deposit continuous films or metal and metal oxide nanoparticles [16,17]. While the deposition of nanostructures into porous structures with pore sizes 50 nm or larger can be easily achieved, it becomes increasingly difficult to uniformly deposit these nanostructures into matrixes with smaller pore sizes [18]. In recent years, various researchers have reported ALD of metal oxide nanoparticles into porous templates, including porous alumina films, transition metal oxide films, mesoporous silica films, SBA-15 and zeolite powders. The pore diameters used ranged from 10 nm to 50 nm in thin films, although there are reports of 3–8 nm pore filling in SBA-15 and zeolite powders [18,19,20,21,22,23,24]. There are no previous reports of ALD into thin film templates with a sub-5 nm pore size. ALD of ZnO is an intensively used and well-established process. The desirable characteristics of ZnO include tunable electrical conductivity, high transparency and piezoelectric properties, resulting in intense interest in its application in solar cells, transistors and sensors [25].

There are limited reports related to the use of mesoporous silica films (MSFs) with pore diameters <10 nm as hard templates for the synthesis of nanostructures. Electrodeposition is mainly employed for the synthesis of metallic nanowires and nanoparticles inside the porous MSFs [5,26,27,28,29]. These MSFs are generally produced using a sol-gel process. A versatile technique known as electrochemically assisted self-assembly (EASA) is also based on a sol-gel process and involves the electrodeposition of MSFs with a pore orientation perpendicular to the electrode surface [30]. The perpendicular orientation of the pores is potentially very advantageous for the synthesis of nanostructures inside these MSFs, as they provide an opportunity for uniform and homogenous pore filling, with one end of the resulting deposit electrically in contact with the electrode used to prepare the MSF.

In this work, we present a facile method based on ALD for the synthesis of zinc oxide (ZnO) nanostructures inside EASA-based mesoporous silica films with sub-5 nm, vertically oriented pores.

## 2. Materials and Methods

Tetraethoxysilane (TEOS, 98%, Alfa Aesar, Leicestershire, UK), ethanol (99.8%, Fisher, Leicestershire, UK), NaNO_3_ (98%, Timstar, Shrewsbury, UK), eicosyltrimethylammonium bromide (C_20_TAB, synthesised in-house [31]), HCl (37%, Fisher, Leicestershire, UK), hexaamine ruthenium (Aldrich, St. Louis, MO, USA) and deionised water (18 MΩ cm, Select Fusion purifier, Willoughby, OH, USA) were used as reagents. Indium tin oxide (ITO) coated on glass (surface resistivity 20 Ω sq^−1^, Ossila Technologies, Sheffield, UK) was used for the working electrodes. The electrochemically assisted self-assembly method was used to prepare mesoporous silica films [30]. Briefly, a sol was prepared using 1:1 *v*/*v* ratio of 0.1 mol dm^−3^ NaNO_3_ and ethanol. A total of 20 mmol dm^−3^ C_20_TAB and 100 mmol dm^−3^ TEOS were added to the solution, and the pH was adjusted to 3 using 0.1 mol dm^−3^ HCl. The sol was stirred for 2 h to hydrolyse. Silica films were electrodeposited on ITO using a three-electrode system, with Ag/AgCl used as the reference electrode and Pt mesh as the counter electrode under potentiostatic conditions (−1.3 V potential applied for 20 s). After electrodeposition, the silica films were immediately rinsed with distilled water and dried at 120 °C for 12 h. Surfactant removal was achieved by dipping the silica films on ITO electrodes in ethanol acidified with HCl for 20 min. Hexaamine ruthenium (1 mmol dm^−3^) was used for electrochemical characterisation of silica films before and after surfactant removal. The MSFs produced using these conditions have a thickness of ~150 nm, with some silica spheres also deposited on their surfaces due to condensation in the bulk solution [30,32].

ALD depositions were performed using a Picosun R200 Advanced deposition tool, Espoo, Finland. Diethylzinc (>95%) was purchased from Strem (Newburyport, MA, USA) and used as received. The diethylzinc and distilled water precursors were held at 18 °C in stainless steel bubblers for all depositions. Purge and line flows used 99.998% N_2_ (150 sccm for diethylzinc, 200 sccm for H_2_O). The pulse and purge times for diethylzinc and H_2_O were 0.1 s and 6.0 s, respectively. All depositions were performed with the chamber temperature set to 220 °C, and film thickness was increased by changing the total number of ALD cycles. During deposition, the reaction chamber was loaded with an MSF sample and with a sample of plain silicon wafer (with ~3 nm of native oxide).

Electrochemical experiments were conducted with a Biologic SP-150 potentiostat, Glossop, UK. A Rigaku Smartlab (Neu-Isenburg, Germany) with Hypix-3000 detector and Cu K_α_ X-rays (λ = 1.54 Å) was used to collect grazing incidence small-angle scattering (GISAXS) and in-plane GISAXS patterns. The distance between sample and detector was 300 mm for GISAXS patterns, and incident angle was adjusted depending upon sample critical angle. In-plane GISAXS patterns were obtained by using the detector in 1D mode. X-ray diffraction data were obtained with the same instrument from 20° to 80° 2θ. The grazing incidence angle was 1°, and the detector was in 1D mode. A Philips XL30 ESEM (Amsterdam, The Netherlands) and a Zeiss Gemini 500 (Oberkochen, Germany) were used to collect scanning electron microscopy (SEM) images and EDX intensity maps. A Jeol ARM 200F (Akishima, Japan) operated at 200 KV was used for transmission electron microscopy (TEM) images. To measure the ALD film thickness on silicon, a J. A. Woollam M2000 Test Base ellipsometer (Quantum Design GmbH, Darmstadt, Germany) was used (angle of incidence 65°). The thickness and refractive index were obtained by using a Cauchy model for fitting ellipsometric data between 450 nm and 1000 nm wavelengths.

## 3. Results and Discussion

### 3.1. Electrochemical Generation and Characterisation of Mesoporous Silica Films

**Figure 1:**
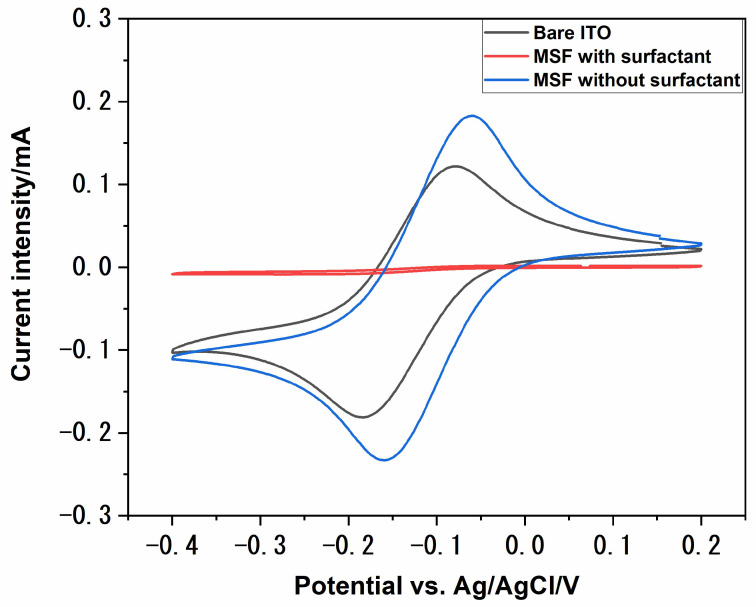
Cyclic voltammograms of 1 mmol dm^−3^ [Ru(NH_3_)_6_]^3+^ in 0.1 M NaNO_3_ on a bare ITO surface (black), MSF with surfactant inside the pores (red) and MSF after surfactant extraction from the pores (blue). Scan rate, 50 mV s^−1^.

Mesoporous silica films were deposited on ITO electrodes using the electrochemically assisted self-assembly (EASA) method. This method is particularly advantageous, as the resulting porous films are homogenous over a wide area on the electrodes, and the pores are oriented perpendicularly to the electrode surface. The film formation process using this method could be divided into the following steps: the potential applied drives the formation of surfactant hemispheric micelles; simultaneously, the applied potential increases the pH of the sol by reducing water to hydroxide; the base-catalysed hydrolysis and condensation of TEOS starts to form a gel around the micelles; and the micelles grow into vertical cylinders as the process continues [33]. These MSFs were further characterised with the help of a redox probe, [Ru(NH_3_)_6_]^3+^. The presence of porous structures on the surface of an electrode can influence the behaviour of redox systems. Mass transport to the underlying electrode surface greatly depends upon the pore diameter, orientation, density and thickness of porous films. Figure 1 shows the cyclic voltammograms recorded at various stages of electrode modifications using 1 mmol dm^−3^ [Ru(NH_3_)_6_]^3+^ in 0.1 mol dm^−3^ NaNO_3_, which was used as a supporting electrolyte. The redox probe had a good reversible behaviour at bare ITO, as expected (black solid line in Figure 1). The electrochemical signal was completely suppressed after film deposition (red solid line in Figure 1), which suggests the deposition of a uniform and defect-free film. The pores are filled with C_20_TAB surfactant at this stage, so the redox probe cannot reach the electrode surface. The redox signal of [Ru(NH_3_)_6_]^3+^ was recovered after surfactant extraction (blue solid line in Figure 1). Surfactant extraction was achieved by dipping the electrode in acidic ethanol and gently stirring. The signal intensity of the MSF–ITO was higher compared to the bare ITO, which is attributed to the accumulation of the redox probe close to the electrode surface due to electrostatic interactions between the positively charged redox probe and the negatively charged pore channels of the MSF [32].

### 3.2. Characterisation of MSF and Deposited Nanostructures by GISAXS and XRD

**Figure 2:**
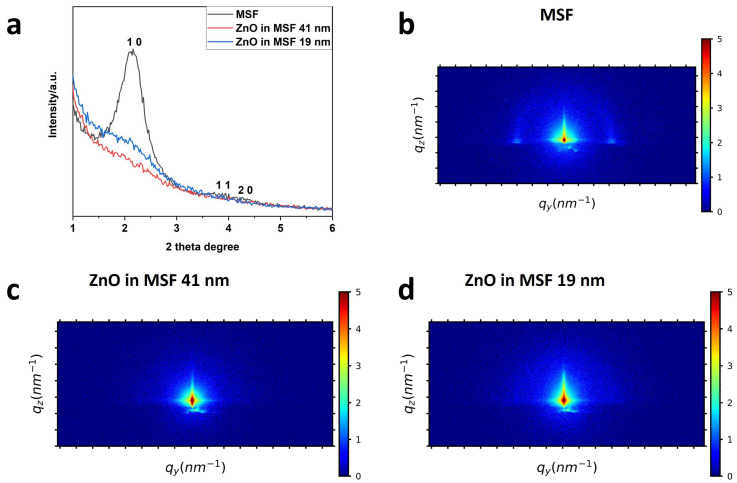
In-plane GISAXS patterns (**a**) of MSF, ZnO 41 nm in MSF and ZnO 19 nm in MSF. The GISAXS patterns of (**b**) MSF, (**c**) ZnO 41 nm in MSF and (**d**) ZnO 19 nm in MSF. The incident angle used for the above patterns is 0.30°.

The mesoporous silica films and ZnO-modified MSFs were characterised using grazing incidence small-angle scattering (GISAXS), as shown in Figure 2. The surfactant extraction was achieved by following the process already in use for similar types of MSFs [33]. The MSF was used after surfactant extraction (open pores), and ZnO deposition was achieved using ALD. The stated thickness of deposition in nm corresponds to that achieved on a flat substrate using the same number of ALD process cycles [34]. These patterns provide information about the mesostructure. The 1 0, 1 1 and 2 0 peaks are visible in the in-plane MSF GISAXS patterns, where the 1 0 peak is more intense than the other two. This indicates hexagonal packing of pores with vertical alignment (perpendicular to the electrode surface). The peak intensities are suppressed after deposition of ZnO, as shown in Figure 2a. We have already shown that in the case of MSF pore filling either by surfactant or nanostructures, the 1 0 peak intensity of in-plane GISAXS patterns is reduced significantly [5]. In Figure 2b–d, the 2D GISAXS images are shown for MSF, ZnO in MSF 41 nm and ZnO in MSF 19 nm, respectively. The two horizontal spots in the MSF pattern correspond to the 1 0 peak and confirm the hexagonal *P*6*mm* mesostructure of vertically oriented pores. The semicircle that is also observed is due to randomly oriented silica spheres deposited on the surface of MSF as a by-product of the electrodeposition process caused by hydroxide diffusing into the bulk solution [32] The disappearance of horizontal spots of ZnO-modified MSFs in Figure 2c,d indicates the pore filling and silica film surface coverage.

**Figure 3:**
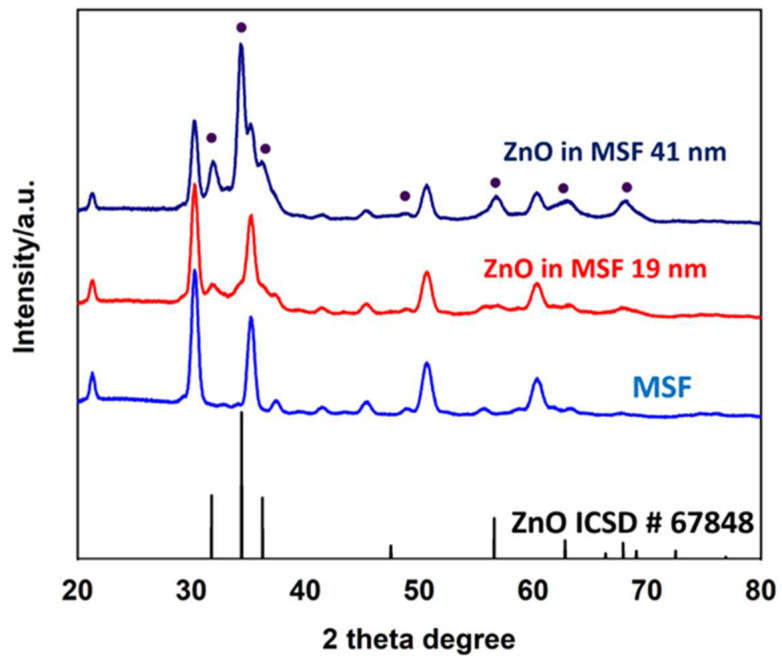
The grazing incidence XRD spectra of MSF, ZnO in MSF 19 nm, ZnO in MSF 41 nm and ZnO (ICSD standard number 67848). The peaks in the MSF sample spectrum arise from ITO coating on the electrode surface.

The grazing incidence X-ray diffraction patterns are shown for MSF, MSF with a 41 nm ZnO coating (MSF 41 nm) and MSF with a 19 nm ZnO coating (MSF 19 nm) in Figure 3. The peaks arising from the MSF sample are from the ITO coating that comprises the working electrode [35]. The peaks for the ZnO in MSF 41 nm and ZnO in MSF 19 nm samples confirm the deposition of polycrystalline hexagonal (wurtzite-type) ZnO [36]. The Miller indices are labelled with reference to a ZnO standard (ICSD 67848). The intensity of ZnO peaks is higher for thicker deposition, as expected.

### 3.3. Microscopic Characterisation of ZnO Modification

**Figure 4:**
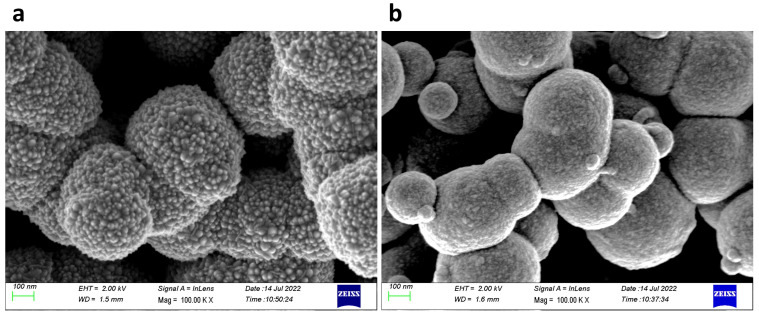
SEM images of (**a**) ZnO in MSF 41 nm and (**b**) ZnO in MSF 19 nm. Images were obtained with a Zeiss Gemini SEM.

**Figure 5:**
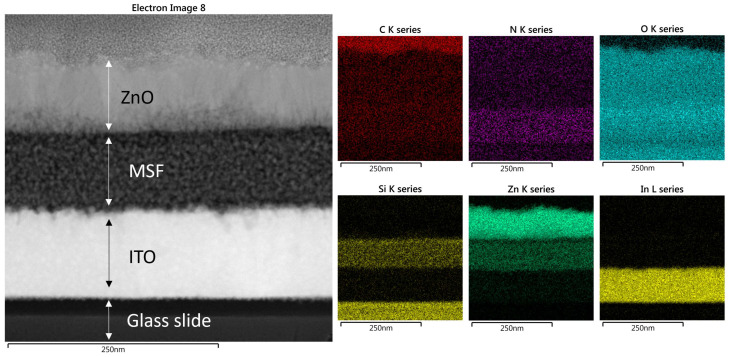
STEM image of ZnO in MSF 41 nm and elemental EDX maps recorded from the same area. The data images were obtained with a Jeol ARM 200-F TEM operated at 200 kV.

Scanning electron microscopy (SEM) images of ZnO in MSF are shown in Figure 4a,b. ALD deploys growth cycles to deposit nanostructures and continuous thin films on different substrates. The images show uniform deposition of ZnO nanostructures on silica spheres. It is evident from the SEM images that a larger number of ALD growth cycles results in a thicker deposition of materials. The spheres seen are present on the MSF surface, and they form as a result of silica condensation during the process of silica film electrodeposition. Figure 5 shows a scanning transmission electron microscopy (STEM) image and corresponding energy-dispersive X-ray spectroscopy (EDX) elemental maps recorded from ZnO (41 nm) in an MSF sample. The ITO-coated glass, silica film and ZnO layer at the top are all visible in the TEM image of the cross-section. The EDX maps clearly indicate that ZnO is deposited inside the porous structure and on the top surface of the MSF. The thick layer of ZnO on the surface is present due to the high number of ALD cycles employed with this substrate.

**Figure 6:**
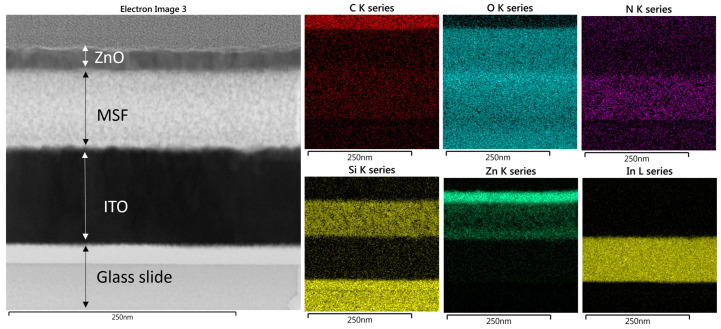
STEM image of ZnO in MSF 19 nm and EDX maps recorded from the TEM image. TEM images were obtained with a Jeol ARM 200-F TEM operated at 200 kV.

The STEM image and corresponding elemental EDX maps for ZnO (19 nm) in MSF are shown in Figure 6, and these are similar to the results with 41 nm ZnO except that a thinner top layer of ZnO is observed at the MSF surface. It is worth noting that, in this case, the thinner layer of ZnO is due to a smaller number of ALD cycles deployed for this sample. It is evident from the EDX maps that nanostructure growth is on the pore walls of the MSF and continues upwards throughout the pores in the form of nanoparticles. Sree et al. showed that the pore size of mesoporous silica films shrinks as a result of increasing ALD cycles, confirming the deposition of nanostructures in the pores. The growth of nanostructures likely starts on the pore walls and on the film surface—at this scale, it is not possible to be clear on whether complete pore filling occurs before the pore entry hole is blocked [24].

**Figure 7:**
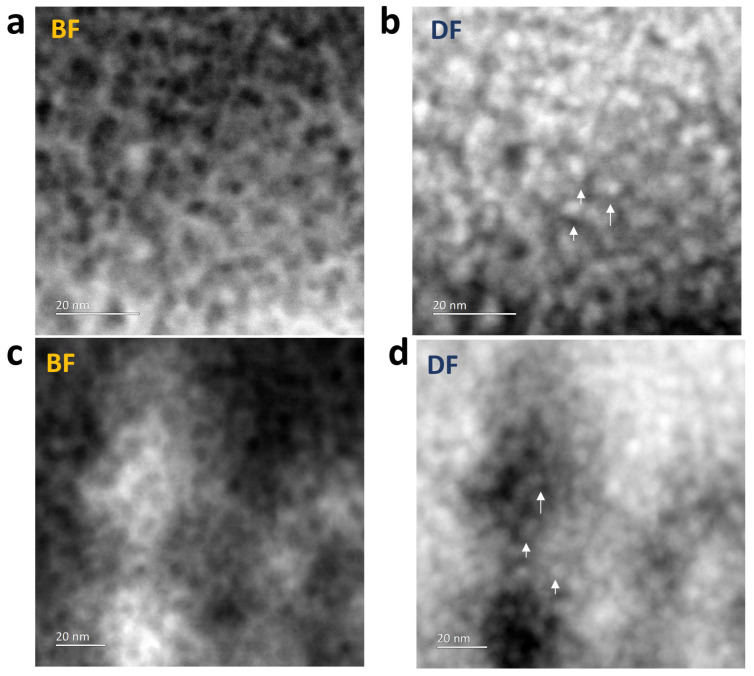
TEM bright- and dark-field images of (**a**,**b**) ZnO in MSF 41 nm and (**c**,**d**) ZnO in MSF 19 nm. The arrows in the dark-field images show that heavier material i.e. ZnO has filled the MSF pores. TEM images were obtained with a Jeol ARM 200F operated at 200 kV.

The microstructure of the ZnO-filled porous silica films was examined by TEM (Figure 7). TEM specimens were prepared by scraping the silica layer off the substrate and spreading the resultant flakes onto lacey carbon films. Figure 6 shows bright-field (BF) and annular dark-field (ADF) STEM images taken from ZnO in MSF 41 and 19 nm, respectively. The bright-field images show the framework of the original porous structure and the dark-field images show that a denser material, i.e., ZnO, fills in the pores.

## 4. Conclusions

Mesoporous silica films with pore diameters of ~4 nm and a vertical orientation were used as templates to deposit ZnO nanostructures using atomic layer deposition. ALD is widely used for the synthesis of nanostructures and thin films that are used in fields such as catalysis, sensing and optics. In this study, we presented a synthesis of ZnO nanostructures in porous hard templates with a uniform sub-5 nm pore size oriented perpendicular to the underlying electrode surface; this method can be easily applied to the synthesis of other metal and metal oxide nanostructures using similar templates. The synthesis of ZnO nanostructures in MSFs was characterised by GISAXS, XRD and electron microscopy.

## Data Availability

The original data presented in the study are openly available in The University of Southampton data repository at https://doi.org/10.5258/SOTON/D3063.
